# Clinical and prognostic differences between manic episodes with and without psychotic symptoms: a 5-year retrospective study with a focus on the non-psychotic presentations

**DOI:** 10.1192/j.eurpsy.2026.10193

**Published:** 2026-07-31

**Authors:** H. Andreu Gracia, A. Giménez-Palomo, L. Olivier, I. Ochandiano, O. de Juan, S. Salmerón, E. Vieta, I. Pacchiarotti

**Affiliations:** 1 Bipolar and Depressive Disorders Unit, Hospital Clinic de Barcelona, Institute of Neuroscience, Hospital Clínic de Barcelona, Barcelona; 2 Centro de Investigación Biomédica en Red de Salud Mental (CIBERSAM), Instituto de Salud Carlos III, Madrid; 3 Institut d’Investigacions Biomèdiques August Pi i Sunyer (IDIBAPS), Barcelona, Spain

## Abstract

**Introduction:**

Psychotic symptoms are frequent during acute episodes of bipolar disorder (BD), particularly in mania, and are traditionally considered a marker of greater severity and worse prognosis. However, data comparing psychotic and non-psychotic mania remain limited and inconsistent.

**Objectives:**

This study aimed to retrospectively examine clinical, therapeutical and 3-year outcome differences between patients hospitalized for mania with and without psychotic symptoms.

**Methods:**

We included all patients admitted for a manic episode to the acute psychiatry unit at Hospital Clínic of Barcelona between 2015 and 2019 (n = 277). Data were extracted from medical records. Patients were followed for 3 years to assess psychiatric emergency department (PED) visits and readmissions. Functional outcomes were also analysed in the BD subgroup (n = 234). Descriptive statistics and survival analyses were used.

**Results:**

Psychotic symptoms were present in 73.6% of the patients and were associated with younger age (p<0.001), earlier onset (p=0.020), poorer insight (p<0.001), higher cannabis use (p=0.018) and manic predominant polarity (p=0006). Non-psychotic patients had more previous admissions for depressive episodes (p=0.003), more previous mixed episodes (p=0.006) and suicide attempts (p=0.002). No significant differences were found in PED visits or readmissions in the next 3 years. Logistic regression identified the number of previous mixed-episode-related admissions as a significant predictor of readmissions (p=0.041), while psychotic symptoms were not associated with either outcome.

**Conclusions:**

While psychotic and non-psychotic mania show clinical and therapeutical differences, psychosis may not predict short-to-medium term outcomes. Non-psychotic mania - which is associated with depressive polarity, mixed features and suicidal behaviour - may indicate a clinically relevant and therapeutically challenging but underrecognized subgroup. These findings call for a nuanced comprehension of the impact of psychosis in mania and multidimensional approaches.

**Disclosure of Interest:**

None Declared

